# Traumatic ventricular septal rupture associated with rapid progression of heart failure despite low Qp/Qs ratio: a case report

**DOI:** 10.1186/s13019-016-0457-4

**Published:** 2016-04-12

**Authors:** Kosuke Murakawa, Susumu Yoshida, Takayuki Okada, Chie Toyoshima, Reisuke Yuyama, Naoki Minato, Ichiro Shiojima

**Affiliations:** Department of Medicine II, Kansai Medical University, Hirakata, Japan; Department of Thoracic and Cardiovascular Surgery, Kansai Medical University, Hirakata, Japan; Department of Cardiology, Rakusai New Town Hospital, Kyoto, Japan

**Keywords:** Ventricular septal rupture, Blunt chest trauma, Low shunt rate, Emergency surgical closure, Early mechanical rupture

## Abstract

**Background:**

Ventricular septal rupture (VSR) secondary to blunt chest trauma is rare and associated with a diverse range of symptoms and clinical courses as well as disease severity. We present a case of traumatic VSR in which rapid progression of heart failure was observed in spite of relatively low pulmonary to systemic blood flow (Qp/Qs) ratio.

**Case presentation:**

A 40-year-old male was transported to the emergency department approximately 12 h after blunt chest trauma. VSR was diagnosed by echocardiography, and right heart catheterization revealed a Qp/Qs ratio of 1.52. Although medical treatment was initially attempted, subsequent rapid progression of heart failure necessitated emergent surgical repair of VSR.

**Conclusions:**

Because small, asymptomatic VSR often close spontaneously, surgical repair of traumatic VSR is indicated when the shunt rate is relatively large or heart failure is present. However, the present case highlights the need to consider emergent surgical repair of traumatic VSR, even when the shunt rate is relatively small.

## Background

Secondary ventricular septal rupture (VSR) is a well-known complication of myocardial infarction. In the current myocardial reperfusion era, incidence rates of VSR complicating myocardial infarction are <1 % [1]. In contrast, secondary VSR following blunt chest trauma is rare. Traumatic VSR is associated with various symptoms and clinical courses as well as disease severity. Although small defects may be treated conservatively, the timing of surgical repair is sometimes disputed. We report a case of traumatic VSR that required emergent surgical repair despite a relatively small shunt ratio.

## Case presentation

**Fig. 1:**
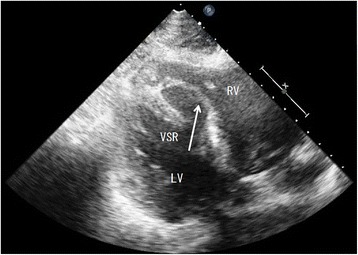
Transthoracic echocardiogram demonstrating the left ventricle (LV), right ventricle (RV), and ventricular septal rupture (VSR)

**Fig. 2:**
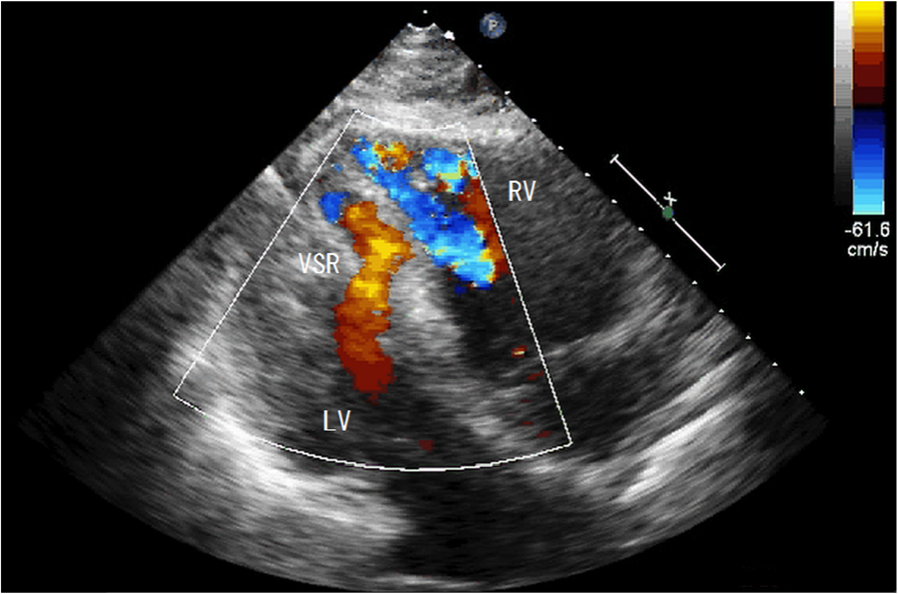
Color Doppler showing left-to-right shunt

A 40-year-old male presented with palpitations immediately after he was struck across the chest during a fight and was transported to the emergency department approximately 12 h after the injury. He did not complain of chest pain. On arrival, his heart rate was 130 beats/min and blood pressure was 115/87 mmHg. A grade 5/6 holosystolic murmur that was most prominent in the fifth intercostal space in the left parasternal region was noted. Chest computed tomography showed no pulmonary contusions, pneumothorax, rib fractures, or pericardial fluid. Creatinine kinase (CK) levels were raised [total CK, 1098 U/L; creatinine kinase with muscle and brain subunits (CK-MB) fraction, 101 U/L]. Electrocardiogram showed sinus tachycardia with a complete right bundle branch block. Transthoracic echocardiograph revealed a longitudinal slit within the ventricular septum and a significant left-to-right shunt (Figs. 1 and 2). The pulmonary to systemic blood flow ratio (Qp/Qs) measured by right heart catheterization was 1.52, and the presence of VSR was confirmed by a significant step-up in blood oxygen saturation at the level of right ventricle. Because the Qp/Qs ratio was relatively small and there were no apparent signs of heart failure, medical treatment was initially attempted. However, approximately 30 h after the injury, he presented with low oxygen saturation and pulmonary edema. Mechanical ventilation, inotropic agents, and an intra-aortic balloon pump were required to maintain the hemodynamic stability of the patient. Despite of these intensive treatments, his blood pressure did not change (95/71 mmHg) and pulmonary arterial pressure gradually increased (from 20 to 38 mmHg). Because a cardiogenic shock state was prolonged, we performed emergent surgical repair on day 2, and VSR was directly closed. Postoperatively the patient remained in heart failure that was refractory to intensive medical treatment, and a residual left-to-right shunt was revealed by echocardiography. We therefore performed a second surgery on day 37, and the defect was closed using a patch. After the second operation, no residual shunt was detected, and the patient’s heart failure completely resolved. The patient was transferred to another hospital for rehabilitation on day 74.

In a series of 207,548 autopsy cases, 546 had nonpenetrating traumatic injury to the heart, among which five cases of isolated VSR were identified [2]. Because of the rarity of this disease condition and the remarkable variability in its severity, the optimal therapeutic strategy for traumatic VSR is not well established. Specifically, the indication of emergent surgical repair of VSR is sometimes disputed. Previous reports suggest that emergent surgery for traumatic VSR should be considered when Qp/Qs exceeds 2.0 or heart failure is present, in part because small VSRs often close spontaneously [3, 4]. The present case however suggests that emergent surgical repair of traumatic VSR should be considered because rapid hemodynamic collapse that necessitates early closure of the VSR can be observed despite a relatively low shunt ratio.

Although the precise mechanism responsible for the development of traumatic VSR is unclear, two possible explanations, early mechanical rupture and delayed inflammatory rupture, are proposed. Early mechanical rupture may occur when a sudden high pressure tears the ventricular septum at the end-diastole when the ventricles are filled and valves are closed [5]. Delayed inflammatory rupture may be caused by defective microcirculation associated with myocardial contusion, leading to necrosis and subsequent rupture of ventricular septum [6]. It is possible that early mechanical rupture causes sudden hemodynamic overload, whereas delayed inflammatory rupture causes gradual hemodynamic changes, leading to more frequent decompensation in patients diagnosed in the early phase. Consistent with this notion, it was previously described that patients with traumatic VSR diagnosed within 48 h after injury were more likely to require emergent surgery and associated with a higher mortality compared with those diagnosed after 48 h of injury [7]. VSR was diagnosed approximately 12 h after injury in the present case, suggesting that VSR was caused by early mechanical rupture and resulted in rapid progression of circulatory failure despite relatively small Qp/Qs ratio. In contrast, in one case report of traumatic VSR in which VSR was diagnosed 11 days after the injury, the patient was discharged without surgical repair, although the shunt ratio in this case (Qp/Qs = 1.52 as measured by echocardiography) was comparable with that of our patient [8]. These observations suggest that special care should be taken when traumatic VSR is diagnosed in the early phase.

Various operative procedures have been developed for VSR. In the present case, the first VSR repair was performed by direct closure through the right ventricle, and the second repair was done by patch closure through the left ventricle. The recurrence of VSR following the first repair is presumably due to direct closure and the collapse of the friable tissue surrounding the site of the direct closure. The risk of VSR recurrence is thought to be relatively high in emergency operations because of the fragility of the myocardial tissues. Thus, the operative approaches and the repair methods for VSR need to be carefully determined in each case, particularly in the acute phase.

## Conclusions

VSR is a rare but serious complication of blunt chest trauma. When VSR is diagnosed in the early phase, the possibility of early progression of heart failure and emergent surgical repair of VSR need to be considered even when the defect is relatively small.

## Consent

Written informed consent was obtained from the patient for the publication of this case report and any accompanying images. A copy of the written consent is available for review by the Editor-in-Chief of this journal.

## Abbreviations

VSR: Ventricular septal rupture
CK: Creatinine kinase
CK-MB: Creatinine kinase with muscle and brain subunits
Qp/Qs: Pulmonary to systemic blood flow ratio
